# Unmasking Long QT Syndrome in the Emergency Department: A Case Report

**DOI:** 10.5811/cpcem.2020.10.48716

**Published:** 2020-12-07

**Authors:** Eric Leslie, Andrew Medenbach, Eric Pittman

**Affiliations:** * United States Naval Hospital Okinawa, Department of Emergency Medicine, Okinawa, Japan; † Naval Medical Center San Diego, Department of Emergency Medicine, San Diego, California

**Keywords:** Long QT syndrome, torsades de pointes

## Abstract

**Introduction:**

Long QT syndrome (LQTS) is an uncommon disorder that can lead to potentially life-threatening dysrhythmias. LQTS can be genetic, acquired, or both.

**Case Report:**

A 44-year-old female with well-controlled hypertension and asthma presented with chest tightness. An initial electrocardiogram yielded a normal corrected QT interval of 423 milliseconds (ms) (normal <480 ms in females). Albuterol was administered and induced agitation, tremulousness, and tachycardia. Follow-up electrocardiograms demonstrated extreme prolongation of the corrected QT interval to 633 ms and morphology change of the T wave. Lab values were later notable for hypokalemia and hypomagnesemia, attributable to a recently started thiazide diuretic. The patient was ultimately diagnosed with congenital LQTS after initial unmasking by albuterol in the emergency department.

**Conclusion:**

LQTS can be unmasked or exacerbated by electrolyte abnormalities and QT prolonging medications.

## INTRODUCTION

Long QT syndrome (LQTS) is a rare condition in which cardiac myocytes are predisposed to repolarization phase abnormalities, which can lead to life-threatening torsades de pointes.^1^ It is one of the leading causes of unexplained sudden cardiac death.^1^ Long QT syndrome can be congenital, acquired, or both.

The prevalence of congenital LQTS is estimated at 1 in 2000 births.^2^ This does not account for patients with electrocardiographically silent LQTS and those who are genotype positive but phenotype negative.^2^ Thus, the true prevalence of the LQTS gene is likely higher. Patients may have electrocardiographically silent LQTS, only to be unmasked by certain QT prolonging medications, electrolyte abnormalities, or sympathetic stimulation.^3^ Type 1 LQTS, one of the most common forms of congenital LQTS, is particularly susceptible to sympathetic stimulation.^3^

Several classes of medications have been demonstrated to lengthen the repolarization phase of cardiac myocytes, thus lengthening the QT interval. Medications classically known to prolong the QT interval include certain antiarrhythmics, calcium antagonists, anti-psychotics, antihistamines, macrolide and fluoroquinolone antibiotics, certain antifungals, and antiretroviral medications (a complete list can be found at crediblemeds.org.).^4^–^6^ In addition, electrolyte abnormalities can similarly affect the repolarization phase. Alterations in serum potassium levels are the most likely to alter the QT interval, however magnesium, calcium and sodium are contributory as well.^4^ Sympathetic stimulation has also been implicated, most notably in sudden exposure to cold water, accounting for a large proportion of sudden cardiac death occurring during swimming.^3^,^7^ In fact, during provocative electrophysiology testing, epinephrine boluses are sometimes used to directly affect the QT interval due to its sympathomimetic effect.^7^ Bradycardia, structural heart diseases, female gender, impaired hepatic and renal function, and advanced age are also known risk factors.^8^,^9^

Due to the above-mentioned risk of sudden death, it is crucially important to recognize LQTS and subsequently follow an appropriate treatment plan. All patients with any significant prolongation of the QT interval, whether transient or persistent, should undergo genetic testing to determine whether they have an underlying channelopathy.^10^ If an underlying genetic alteration is identified, family members should be counseled on the need for further testing and evaluation.^10^ Treatment for patients with acquired QT prolongation includes withholding medications with known QT prolonging effects and avoiding serum electrolyte perturbations. Treatments for patients with congenital prolonged QT include the above strategies, observation, beta blocker therapy, avoidance of high intensity sports, or implantable cardiac defibrillator (ICD) placement.^11^

## CASE REPORT

A 44-year-old female with a history of well-controlled asthma and hypertension presented to the emergency department (ED) with “chest tightness”, which the patient described as inability to take a full breath with occasional pressure-like sensation across the precordium. The patient denied exertional component, leg swelling, history of coagulopathy, thrombotic risk factors, family history for coronary artery disease, cough, fever, vomiting, or abdominal pain. One week prior to ED presentation, the patient had a routine checkup with her family physician and was started on chlorthalidone for her hypertension. She was prescribed the following home medications: montelukast, fluticasone, chlorthalidone, loratidine, ascorbic acid, ferrous sulfate, and albuterol.

The patient’s initial presenting vital signs were the following: pulse 82 beats per minute; blood pressure of 123/73 millimeters of mercury; respirations 18 per minute; oxygen saturation 100%; temperature 98.2 degrees Fahrenheit. On physical exam, breath sounds were mildly diminished in all lung fields. A subtle end expiratory wheeze was appreciated. There was no accessory muscle use or increased respiratory effort. There was no evidence of stridor or upper airway swelling. Cardiac auscultation demonstrated a regular rate and rhythm with no murmur appreciated. The abdomen was soft, nontender and nondistended. Pulses were equally present and strong in all four extremities. No lower extremity swelling was appreciated. A chest radiograph was within normal limits. An initial electrocardiogram (ECG) (Image A) demonstrated no acute ischemia or dysrhythmia (Bazett QTc 423 milliseconds [ms]).

A troponin was similarly negative after days of symptoms, lowering concern for acute coronary syndrome. A trial of albuterol was instituted as some of her symptoms were considered to be attributable to her otherwise well-controlled asthma. Shortly after receiving the nebulized albuterol treatment, the patient became tachycardic and very tremulous. A repeat ECG was obtained (Image B) and notable for a significantly prolonged QT interval (Bazett QTc 633 ms) with distinct morphology change of the T wave. Laboratory studies were reviewed and notable for hypokalemia of 3.2 milliequivalents per liter (mEq/L) (reference range 3.4–5.1 mEq/L) and hypomagnesemia of 1.6 milligrams per deciliter (milligrams per deciliter [mg]/dL) (reference range 1.7–2.2 mg/dL). The albuterol treatment was terminated. The patient was immediately treated with 2 grams intravenous (IV) magnesium sulfate, 20 mEq IV potassium chloride, and 40mEq oral potassium chloride. After one hour, the QT interval had shortened (QTc 428 ms) (Image C) but retained the T wave morphology change. The patient was observed in the ED for several more hours, and discharged home with a next day follow-up to have her electrolyte levels reassessed. The patient’s chlorthalidone and albuterol were discontinued.

**CPC-EM Capsule**

What do we already know about this clinical entity?

Long QT Syndrome can predispose patients to Torsades de Pointes. Certain medications are known to further prolong the QT interval.

What makes this presentation of disease reportable?

The patient’s underlying Long QT syndrome was effectively unmasked in the emergency department (ED) in the context of albuterol treatment and electrolyte changes from diuretic use.

What is the major learning point?

Be vigilant of the QT prolonging effects of certain medications and electrolyte derangements to further prolong the QT interval. Genetic Long QT Syndrome may only be “unmasked” briefly.

How might this improve emergency medicine practice?

Genetic Long QT Syndrome can be identified in the ED and potentially life-saving referrals can be made.

The patient was referred to an electrophysiologist and ultimately diagnosed with Type 1 LQTS. The patient’s family was referred for genetic counseling. Despite the patient’s asthma being a contraindication, after a risk-and-benefit conversation she was initiated on low-dose beta blocker therapy which was well tolerated. She declined placement of an ICD. The importance of close monitoring of her electrolytes was stressed, particularly during situations in which electrolyte loss is possible (diarrhea, vomiting, exercise).

## DISCUSSION

This case was unique in that it contained elements of both genetic and acquired LQTS. Given her normal ECG at presentation, this represents a case of electrocardiographically silent LQTS. Only after provocation with albuterol did she have demonstrable prolonged QT on ECG. In addition, the electrolyte abnormalities caused by the chlorthalidone lowered the threshold for abnormalities in the repolarization phase. The computer-calculated QTc should always be checked with a manual QTc. This can be done by using Bazett’s equation. This should be repeated with multiple different beats in different leads, preferably lead II and V5. After correction the patient’s QT was calculated to be 633 ms. See Image 1 for our calculation.

Patients with congenital LQTS most commonly have mutations to the Kv11.1 and Kv7.1 potassium channel proteins, responsible for the rapid (I_KR_) and slow (I_KS_) delayed potassium rectifier currents, respectively.^8^ These altered I_KR_ and I_KS_ currents can lead to excessive local extracellular potassium levels and ultimately repolarization abnormalities.^12^ Should these gradients worsen, the patient is at risk for entering torsades de pointes.

The figure is a schematic demonstrating the action potential of a cardiac myocyte.^13^ As described above, I_KR_ and I_KS_ are the most commonly affected currents in congenital LQTS. These will directly affect phase three, causing a delay in repolarization. On the ECG, this corresponds to the altered T wave morphology and subsequently the prolonged QT interval.

In addition, generalized sympathetic stimulation can prolong the QT interval. In fact, in electrophysiology laboratory testing, patients are given epinephrine boluses to elicit sympathetic stimulation to unmask LQTS.^3^ The albuterol “challenge” that was administered in the ED certainly could have had a similar sympathomimetic effect. Indeed, a study by Thottathil et al showed beta-2 agonist use by patients with LQTS is a risk factor for cardiac events.^14^ Asthmatic patients with LQTS who need beta-2 agonists should have electrolyte levels monitored and repleted as necessary. When not contraindicated, the provider should also consider using a beta blocker as there is decreased risk of cardiac events when beta blockers are used.^14^

## CONCLUSION

In conclusion, this is a cautionary tale in which a patient with an underlying cardiac channelopathy was administered a QT prolonging agent in the context of multiple electrolyte abnormalities induced by a recently started thiazide diuretic. Caution should be exercised in administering patients a medication with known QT prolonging effects. It is not infrequent that several medications with the potential for QT prolongation may be used simultaneously in the ED setting (eg, an agitated elderly patient with chronic obstructive pulmonary disease found to have pneumonia). In patients receiving these medications, consider first obtaining an ECG and/or placing the patient on a cardiac monitor.

## Figures and Tables

**Figure:**
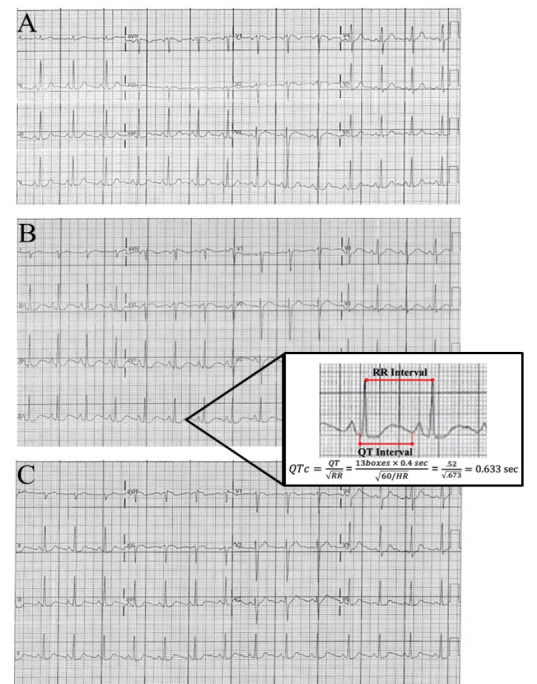
Cardiac Myocyte Action Potential. Slow delayed rectifier potassium current (I_KS_) and rapid delayed potassium rectifier current (I_KR_) are responsible for efflux of potassium ion during the repolarization phase of the cardiac myocyte (Phase 3). Abnormal serum potassium levels can further affect repolarization in the context of dysfunctional potassium channels. *K*^+^*,* potassium; *Na*^+^*,* sodium; *Cl*^−^*,* chloride; *Ca^2+^,* calcium; *I_K1_,* inward rectifier potassium current; *I_Na_,* inward sodium channel; *I_to1,2_,* transient outward potassium current; *I_Ca-L_,* L-type calcium current; *I_KS_,* slow delayed rectifier potassium current; *I_KR_,* rapid delayed potassium rectifier current; *ms,* milliseconds. *Adapted and modified from Wikimedia Commons with permission.

**Image:**
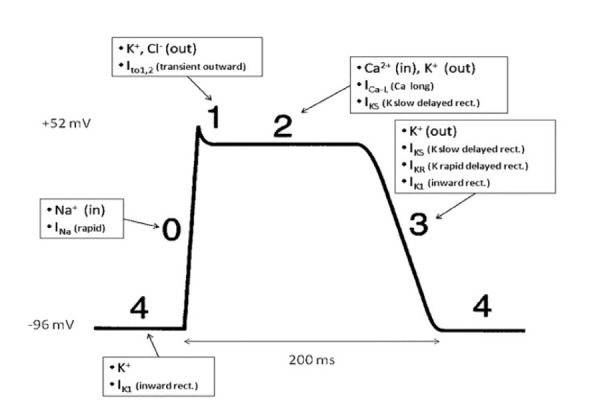
Serial electrocardiograms (ECG) demonstrating QT prolongation after albuterol use. ECG A demonstrates the patient’s baseline with no acute dysrhythmia or ischemia and QTc of 423 milliseconds (ms). ECG B demonstrates a QTc interval of 633 ms and T wave morphology change. ECG C demonstrates QTc return to 428ms, with retention of the T wave morphology.
